# Alternative Therapy for Epstein-Barr Virus Related Hemophagocytic Lymphohistiocytosis

**DOI:** 10.1155/2015/508387

**Published:** 2015-02-08

**Authors:** Omar Al Asad, Amir Salam, Siva Mannem, Mary Ninan, Avi Markowitz, Bagi Jana

**Affiliations:** Division of Hematology and Oncology, Department of Internal Medicine, University of Texas Medical Branch, 301 University Boulevard, Galveston, TX 77555, USA

## Abstract

Hemophagocytic lymphohistiocytosis (HLH) is a rapidly fatal condition characterized by excessive immune activation. HLH can occur as a familial or sporadic acquired disorder. Acquired HLH is more frequently found in adults and is commonly secondary to infections, malignancies, or autoimmune diseases. Diagnosing HLH is challenging because of the rare occurrence, variable presentation, and nonspecific findings of this disorder. Diagnosis of HLH can be based on the diagnostic criteria which were used in the HLH-2004 trial. Given the rarity of this disease, protocols for its treatment have developed slowly, and obtaining adequate short-term and long-term control of the disease continues to be a challenge. Conventional induction therapy for HLH is dexamethasone and etoposide (VP-16), followed by or with cyclosporine. Intrathecal methotrexate ± hydrocortisone is given to those with central nervous system disease. We are reporting a patient who was diagnosed with Epstein-Barr virus (EBV) related HLH. He achieved complete remission with rituximab alone. To our knowledge, this is the first case of an adult patient with EBV related HLH who went into remission with rituximab therapy alone, without using the conventional chemotherapy.

## 1. Introduction

Hemophagocytic lymphohistiocytosis (HLH) is a rapidly fatal condition characterized by fevers, hepatosplenomegaly, and cytopenias. The disease is considered to be a syndrome of excessive immune activation [1]. HLH can occur as a familial or sporadic disorder.

Acquired HLH is more frequently found in adults and is commonly secondary to infections, malignancies, or autoimmune diseases.

Diagnosing HLH is challenging because of the rare occurrence, variable presentation, and nonspecific findings of this disorder. Although the individual signs and symptoms of HLH may occur in a variety of clinical circumstances, the combination of these features, caused by pathologic inflammation, forms the pattern of HLH. Diagnosis of HLH can be based on the diagnostic criteria which were used in the HLH-2004 trial [2, 3] as follows:

A. molecular diagnosis consistent with HLH: pathologic mutations of PRF1, UNC13D, Munc18-2, Rab27a, STX11, SH2D1A, or BIRC4, or

B. five of the 8 criteria listed below which are fulfilled:

1. fever ≥ 38.5°C;
2. splenomegaly;
3. cytopenias (affecting at least 2 of 3 lineages in the peripheral blood);
   hemoglobin < 9 g/dL (in infants < 4 weeks: hemoglobin < 10 g/dL),
     platelets < 100 × 10^3^/mL,
     neutrophils < 1 × 10^3^/mL;
4. hypertriglyceridemia (fasting, >2.9 mmol/L) and/or hypofibrinogenemia (<4.4 mmol/L);
5. hemophagocytosis in bone marrow, spleen, lymph nodes, or liver;
6. low or absent NK-cell activity;
7. ferritin > 1124 mmol/L;
8. elevated sCD25 (*α*-chain of sIL-2 receptor).

Given the rarity of this disease, protocols for its treatment have developed slowly, and obtaining adequate short-term and long-term control of the disease continues to be a challenge.

EBV is the most frequent infection associated with HLH [2]. Because it can eliminate EBV-infected B cells, rituximab may be a beneficial addition to other therapies in patients with progressive EBV-HLH [4]. We present in this report a case of a patient who was diagnosed with HLH after meeting the diagnostic criteria according to HLH-2004. He achieved complete remission with rituximab alone, weekly for 4 doses.

## 2. Case Report

**Figure 1:**
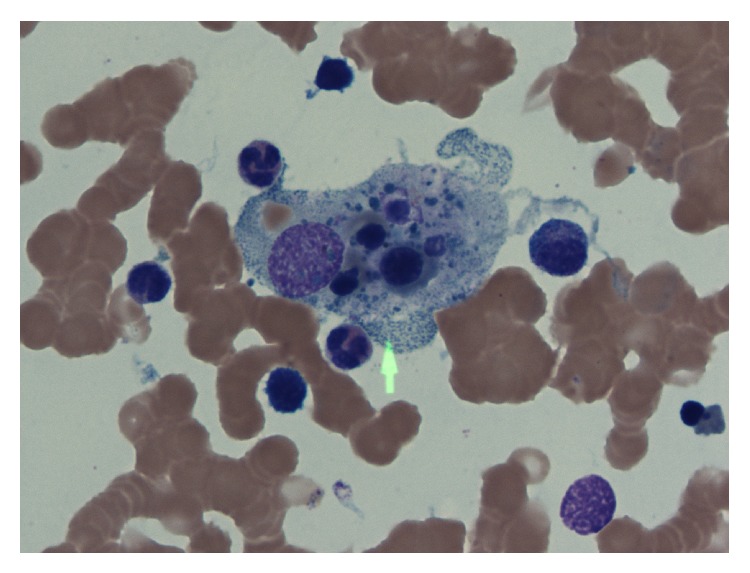
The histiocyte in the center of this picture appears “stuffed” with engulfed erythrocyte precursors.

A 52-year-old male presented to the hospital with 2 months of fevers of up to 40 degrees Celsius, weakness, and a 15 kg weight loss over 6 months. His past medical history was notable for a cadaveric renal transplant of over 20 years for which he had been on immunosuppressant therapy, mycophenolate, sirolimus, and prednisone. On admission, he was found to have profound anemia with hemoglobin of 4.9 g/dL, a platelet count of 50,000/uL, and ferritin above 22470 mmol/L. He also had elevated liver transaminases fluctuating between 100 and 200 mmol/L. A bone marrow biopsy was performed to look for other causes of his cytopenias such as infectious infiltration; he was found to have hemophagocytosis involving 50% of the histiocytes (Figure 1). Further workup revealed a triglyceride level of 9.04 mmol/L, LDH of 2000 mmol/L, and a soluble IL-2 level of 23,690 pg/mL. Given his history of being on immunosuppressants, an infectious workup for opportunistic infections was investigated upon admission to the hospital. All microbiological cultures were negative. However, he was later found to have profound Epstein-Barr virus infection with 9,380,000 copies/mL in his peripheral blood, as well as EBV positivity in his cerebrospinal fluid. CT of chest, abdomen, and pelvis did not show masses or lymphadenopathy, so we ruled out posttransplant lymphoproliferative disorder. The diagnosis of HLH was made as he met the diagnostic criteria: fever, hemophagocytosis on bone marrow biopsy, elevated ferritin, cytopenias, hypertriglyceridemia, and high soluble IL-2 level.

The patient's outpatient immunosuppressants were held. He was begun on dexamethasone 10 mg/m^2^/day according to the HLH-94 protocol [2]. After being on therapy for 3 days, his fevers and cytopenias did not improve. Due to his abnormal kidney and liver function, we did not start conventional chemotherapy (etoposide) [2] and gave him instead rituximab weekly for the EBV infection (375 mg/m^2^ intravenously). This was based on the knowledge regarding the relationship between EBV infections and HLH [2].

**Figure 2:**
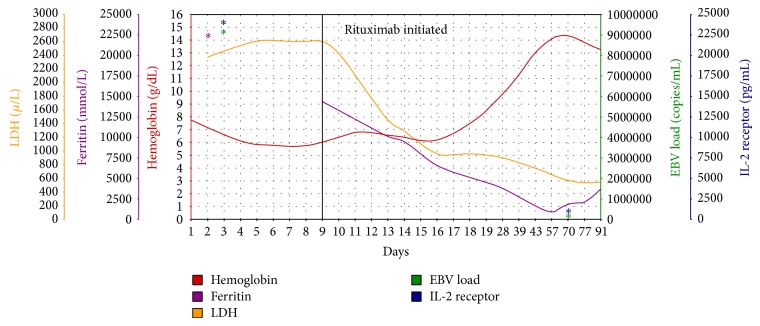
Trend of hemoglobin and markers of disease severity before and after starting rituximab.

Within one week following the first dose, his fevers, cytopenias, liver function tests, and inflammatory markers improved significantly. He was given his second dose of rituximab in the hospital setting and discharged safely to continue his third and fourth doses of rituximab in the outpatient setting. During 3-month follow-up period, the patient remained in complete remission. His counts normalized with the exception of his hemoglobin, as he remained mildly anemic due to his transplanted kidney failure and the initiation of dialysis. EBV viral load was checked 7 weeks after finishing treatment, and it was undetectable (Figure 2).

## 3. Discussion

The goal of therapy for patients with HLH is to suppress life-threatening uncontrolled inflammatory reaction. Conventional induction therapy for HLH is dexamethasone and etoposide (VP-16), followed by or with cyclosporine. Intrathecal methotrexate ± hydrocortisone is given to those with central nervous system disease [2, 5].

There have been studies on the treatment of Epstein-Barr virus-induced haemophagocytic lymphohistiocytosis with rituximab-containing chemoimmunotherapeutic regimens. These studies demonstrated that rituximab-containing regimens significantly reduce EBV load and signs of inflammation [6, 7].

To our knowledge, this is the first case of an adult patient with EBV related HLH who went into remission with rituximab therapy alone. On review of the literature, we did find a case in which an adolescent with Crohn's disease was treated with rituximab and had good response [8]. Some case reports also showed the effectiveness of rituximab alone in treating HLH secondary to different triggers other than EBV [9, 10].

Rituximab is chimeric monoclonal antibody against the protein CD20. By targeting CD-20 positive B-cells, rituximab decreases the load of the causative pathogen (EBV), which in turn decreases the EBV-induced hyperactive immune response.

In conclusion, rituximab is a potential alternative treatment for EBV related HLH patients who are not fit for conventional chemotherapy with etoposide. Further clinical trials are warranted on rituximab as a sole treatment.
